# Effects of Natural Polyphenols on Skin and Hair Health: A Review

**DOI:** 10.3390/molecules27227832

**Published:** 2022-11-14

**Authors:** Mang Sun, Ya Deng, Xining Cao, Lu Xiao, Qian Ding, Fuqing Luo, Peng Huang, Yuanyuan Gao, Mengqi Liu, Hengguang Zhao

**Affiliations:** 1 Department of Dermatology, The Second Affiliated Hospital of Chongqing Medical University, Chongqing 400010, China; 2 Department of Dermatology, Daping Hospital, The Army Medical University, Chongqing 400042, China; 3 Bioengineering College, Chongqing University, Chongqing 400030, China

**Keywords:** polyphenols, skin, hair, human health

## Abstract

The skin is the largest organ of the body and plays multiple essential roles, ranging from regulating temperature, preventing infections, to ultimately affecting human health. A hair follicle is a complex cutaneous appendage. Skin diseases and hair loss have a significant effect on the quality of life and psychosocial adjustment of individuals. However, the available traditional drugs for treating skin and hair diseases may have some insufficiencies; therefore, a growing number of researchers are interested in natural materials that could achieve satisfactory results and minimize adverse effects. Natural polyphenols, named for the multiple phenolic hydroxyl groups in their structures, are promising candidates and continue to be of scientific interest due to their multifunctional biological properties and safety. Polyphenols have a wide range of pharmacological effects. In addition to the most common effect, antioxidation, polyphenols have anti-inflammatory, bacteriostatic, antitumor, and other biological effects associated with reduced risk of a number of chronic diseases. Various polyphenols have also shown efficacy against different types of skin and hair diseases, both in vitro and in vivo, via different mechanisms. Thus, this paper reviews the research progress in natural polyphenols for the protection of skin and hair health, especially focusing on their potential therapeutic mechanisms against skin and hair disorders. A deep understanding of natural polyphenols provides a new perspective for the safe treatment of skin diseases and hair loss.

## 1. Introduction

The skin is the largest organ, with a complicated structure and multiple physical functions, thus providing a barrier against outside hazards. In addition, the skin is also involved in regulating the hydroelectrolytic balance and immune response of individual organisms. Due to the extensive distribution and functional diversity of the skin, skin diseases are among the most common disorders. A hair follicle is a complex cutaneous appendage. Studies have shown that hair loss has a significant effect on the quality of life and psychosocial adjustment of people. Hair loss can lead to social anxiety, symptoms of depression and anxiety, low self-confidence, and dissatisfaction with life [1]. Many factors are related to skin and hair diseases, including genetics, local infections, endocrine disorders, and mental stress. People have used various drugs and remedies to treat skin and hair diseases according to their different pathogeneses. However, the available drugs for treating skin and hair diseases still have many drawbacks. Considering the occurrence and complexity of skin and hair diseases, as well as the adverse effects of available drugs, research investigating novel remedies and less dangerous natural materials has increased in recent years.

**Figure 1:**
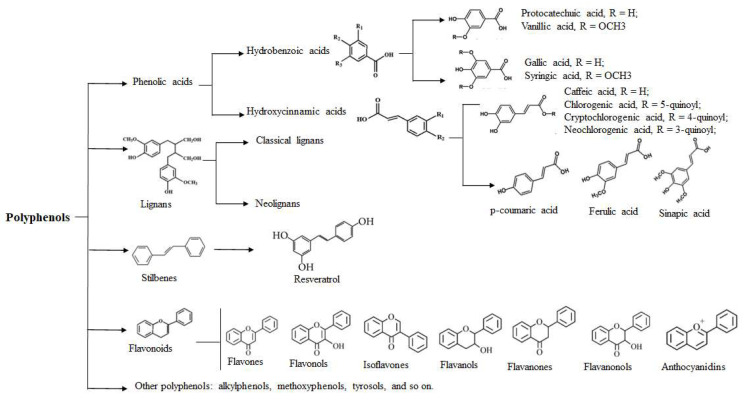
Main classification and basic chemical structures of polyphenols.

Polyphenols, which are widely found in plants, play an increasingly important role in protecting human health. Polyphenolic compounds are chemical substances commonly found in fruits, vegetables, and cereals. They are named for the multiple phenolic hydroxyl groups in their structures. In recent years, along with developments in science and technology that have enabled structure identification, over 1000 kinds of polyphenols have been identified, and their pharmacological activities have been extensively studied. According to their different chemical constitutions, polyphenols are mainly classified into four groups, including phenolic acids, flavonoids, stilbenes, and lignans (Figure 1). Phenolic acids can be further identified as hydroxybenzoic and hydroxycinnamic acids. Some well-known polyphenols include resveratrol, quercetin, curcumin, epigallocatechin gallate, catechin, hesperetin, cyanidin, procyanidin, caffeic acid, and genistein [2]. Polyphenols have a wide range of pharmacological effects. In addition to the most common effect, antioxidation, polyphenols have anti-inflammatory, bacteriostatic, antitumor, and other biological effects associated with reduced risk of a number of chronic diseases, including cardiovascular disease and cancer [3,4,5,6]. Various polyphenols have also shown efficacy against different types of skin and hair diseases, both in vitro and in vivo, via different mechanisms [7,8,9]. Herein, we primarily focused on the effects and mechanisms of polyphenols related to skin and hair health.

## 2. Roles of Polyphenols in Skin Health

### 2.1. Anti-Inflammatory Effects of Natural Polyphenols 

Inflammatory skin diseases encompass a wide spectrum of skin disorders and affect people of all ages and skin types. The majority of chronic inflammatory skin diseases manifest a relapsing and remitting course throughout life, including atopic dermatitis, psoriasis vulgaris, lichen planus, and so on. These diseases are associated with complex multifactorial etiologies in which genetic and environmental factors interact both in the genesis and development of the disease. Specifically, signaling molecules released from the injured stratum corneum initiate a cytokine cascade, triggering an inflammatory response, which then contributes to the pathogenesis of a variety of dermatoses [10]. Glucocorticoids and biological agents are now commonly used to manage inflammatory skin diseases via different mechanisms, but systemic corticosteroids and immunosuppressives can only be used for short-term treatment because of their serious adverse effects, including growth inhibition, hematopoietic suppression, glaucoma, hypertension, hyperglycemia, osteoporosis, myopathy, cataracts, infection, and thin or easily bruised skin [11]. Biological therapies have revolutionized moderate-to-severe inflammatory dermatosis treatment, focusing on inhibiting selective key pathways of inflammation, including interleukin-4 (IL-4), IL-13, IL-31, IL-12/23, IL-17, thymic stromal lymphopoietin (TSLP), and tumor necrosis factor (TNF-α) [12]. Side effects of biological agents remain unavoidable, for instance, associated serious bacterial, viral, and fungal infections, including active hepatitis B virus, reactivation of latent tuberculosis infection, and increased risk of *Candida* infections, as well as worsening of pre-existing inflammatory bowel disease and, rarely, new-onset ulcerative colitis [13,14,15,16].

Many polyphenols, especially flavonoids, possess potent anti-inflammatory properties and can regulate immunity [17,18,19,20,21]. Several natural polyphenols have been well studied for their beneficial effects in autoimmune inflammatory diseases. Some polyphenols, such as resveratrol, chlorogenic acid, caffeic acid, pelargonin, and ferulic acid, modulate pro-inflammatory gene expression and cytokine production, thus impacting immune cell populations [22,23]. The non-flavonoid curcumin was shown to downregulate the expression of TNF, IL-1, adhesion molecule-like vascular cell adhesion molecule-1 (VCAM-1), and intercellular adhesion molecule-1 (ICAM-1) in human umbilical vein endothelial cells and inflammatory mediators such as prostaglandins and leukotrienes. Topical application of green tea polyphenols (GTPs) and epigallocatechin-3-gallate (EGCG) resulted in inhibited production of prostaglandin metabolites, including prostaglandin D2 (PGD2), prostaglandin E2 (PGE2), and prostaglandin F2α (PGF2α) [24]. Resveratrol can induce endothelial nitric oxide synthase (eNOS), inhibit cyclooxygenase (COX), and inactivate peroxisome proliferator-activated receptor gamma (PPARγ) in vitro and in vivo [25,26]. What’s more, curcumin downregulated signal transducer and activator of transcription 3 (STAT3) and nuclear factor kappa-light-chain-enhancer of activated B cells (NF-κB) and reduced the expression of toll-like receptor-2 (TLR-2) and -4 while upregulating PPARγ in an in vivo study [27,28]. Caffeic acid phenethylester suppresses LPS-mediated TLR-4 and NF-κB activation in macrophages. Quercetin was also confirmed to inhibit leukotriene biosynthesis in human polymorphonuclear leukocytes [29].

Based on their anti-inflammatory and immunomodulatory effects, natural polyphenols are used to treat a variety of skin diseases. Vitiligo is a common skin disorder characterized by hypopigmentation. *Ginkgo biloba* is known to be a rich source of polyphenolics. *G. biloba* extract was associated with the progression of vitiligo by reducing depigmentation and promoting repigmentation [30,31]. Carnosic acid is a natural benzenediol abietane diterpene found in rosemary. Carnosol was able to reduce levels of neutrophils, inflammatory cytokines (IL-1β and TNF-α), COX-2, and iNOS in mice blood [32,33]. Animals with atopic dermatitis topically treated with carnosol showed obvious skin lesion reductions [34]. Artichoke polyphenols, as potential anti-inflammatory agents, can improve the vasodilatation and microcirculation of endothelial cells by inhibiting nitric oxide (NO) production in both macrophages and endothelial cells. Moreover, artichoke polyphenols can improve skin elasticity and roughness by inhibiting vascular aging, thus acting as a protective ingredient for both lymphatic and endothelial cells. These effects could be the direct result of their antioxidant or anti-inflammatory properties and indirect result via modulation of molecular pathways that improve the expression of genes involved in anti-aging mechanisms [35]. 

### 2.2. Antioxidant Properties of Natural Polyphenols

Human life is dependent upon oxygen. Occasionally, oxygen becomes mutagenic and toxic. Oxidative stress plays a very important role in human dermal diseases and skin aging [36,37]. Overproduction of reactive oxygen species (ROS) can damage the membranes, lipids, proteins, RNA, and DNA of cells. The traditional view is that the antioxidant activity of a polyphenol is positively correlated with its number of phenolic hydroxyl groups. As excellent antioxidants, the phenolic hydroxyl groups of polyphenols can decrease levels of free radicals by providing electrons and can also be used as free radical scavengers or metal-chelating agents (chelating metals with redox activity, such as copper and iron) to inhibit or eliminate the formation of free radicals, thereby destroying the progress of free radical chain peroxidation [38]. 

The skin is directly and frequently exposed to ultraviolet (UV) rays from the sun (UVA: 320–400 nm and UVB: 280–320 nm) [39,40]. UV radiation is involved in the pathogenesis of severe skin conditions, including photoaging of the skin, immune disorders, and skin cancer [41,42]. Many plants are rich in antioxidants because they must survive continual UV radiation exposure. For example, a marine algal polyphenol isolated from the brown alga *Ecklonia cava* was confirmed to have an inhibitory effect on melanogenesis and a protective property against photo-oxidative stress induced by UVB. Intracellular ROS induced by UVB radiation was reduced by the addition of the marine algal polyphenol and cell viability was dose-dependently increased. Moreover, the marine algal polyphenol demonstrated strong protective properties against UVB radiation-induced DNA damage, including damaged tail intensity and morphological changes in fibroblasts [43]. Clove is another kind of plant that is widely used in Chinese medicine and also used in the cosmetics industry. Cloves are rich in natural polyphenols such as ferric acid. Our previous study proved that cloves can decrease UVB damage through their influence on Na^+^-K^+^-ATPase, which led to a reduction in oxidation and inflammation in mice, thereby inhibiting skin injury and protecting the skin [44]. The above studies demonstrate that many natural polyphenols contribute to the prevention of UVB skin damage and inhibit photodamage to the skin. They are very promising for future research and applications.

Estrogen deficiency is associated with deteriorating skin health as it affects internal structural balance, dermal cellular mechanisms, and other biological functions. The effects of estrogen deficiency include loss of elastin, collagen, fibroblast dysfunction, increased vascular and matrix metalloproteinase activity, and extracellular and cellular degradation, leading to wrinkles, atrophy, dryness, impaired wound healing/barrier function, and reduced antioxidant capacity. Several studies have examined polyphenolic phytochemicals, also known as phytoestrogens, which act as estrogen receptor modulators (SERMs) and possess ERβ-agonist properties [45]. The resveratrol compound extracted from grapes has been known for its anti-aging effects for over a decade [22,46]. Recently, an increasing number of studies have reported the benefits of resveratrol on the skin, including its antioxidant properties, which are achieved through activation of nuclear factor erythroid 2-related factor 2 (Nrf2) by reducing the expression of nuclear factor kappa-B (NF-кB) and activating protein 1 (AP-1), fibroblast proliferation by increasing type I, II, and III collagen expression through activation of sirtuin 1 (SIRT 1, anti-aging factor), and inhibition of melanogenesis [45,46,47]. In preliminary studies, human skin benefited from several types of resveratrol analogs, the most potent of which was 4’-acetoxyresveratrol (4AR) [45,48], which increased human genetic expression of the antioxidant superoxide dismutase (SOD) [45]. Equol is a relatively new phytochemical found in food sources and plants [45,49,50]. It is classified as a phytoestrogen with selective SERM properties and binds to ERβ in keratinocytes [49,50,51]. Equol exhibits skin-protecting antioxidant properties. In a clinical investigation involving a 12-week single-center study with 59 female subjects, equol significantly improved skin characteristics, including hydration and firmness, which suggested that equol may be effective in treating estrogen-deficient skin [52]. 

Along with their antioxidant properties, some natural polyphenols have potential whitening effects and prominent protective effects against cell damage and skin aging, which may be used in the cosmeceutical and pharmaceutical industries (Table 1) [44,53,54,55,56].

### 2.3. Anti-Allergic Effects of Natural Polyphenols

It is said that allergic diseases are prevalent in approximately 40% of the general population and will rapidly increase to 50% [57,58]. The skin is very often the target organ involved in allergic reactions, including urticaria, angioedema, atopic dermatitis, contact dermatitis, and vasculitis. This may be associated with many immunologically competent cells, such as mast cells, lymphocytes, eosinophils, neutrophils, and Langerhans cells, especially antigen-presenting Langerhans cells [59]. As an alternative to conventional treatments with corticosteroids and antihistamines, polyphenols also exhibit anti-allergic effects, including inhibiting the production of proinflammatory cytokines and leukocytes, as well as histamine release [60]. Polyphenols have also been shown to regulate the balance of Th1/Th2 and inhibit the formation of antigen-specific IgE antibodies. Two main mechanisms may be involved in this process. Firstly, polyphenols may affect the allergen-IgE complex formation [61]. Secondly, polyphenols may affect the binding of this complex to its receptors (FceRI) on basophils and mast cells [62]. For instance, the ingestion of tannins extracted from apples has been proven to prevent food allergies, which may be associated with the increased proportion of γδ TCR T cells in intestinal intraepithelial lymphocytes [63]. EGCG has a strong suppressive effect on the migratory and adhesive abilities of peripheral blood B cells. This suppressive effect is mediated by the binding of EGCG to CD11b on B cells, and the consequent suppression of B-cell extravasation to the extravascular space. Because of the important role played by B cells in humoral immunity, EGCG is a promising drug for the prevention and/or treatment of skin allergic diseases [64]. Overall, polyphenols hold promise as anti-allergy agents capable of influencing multiple biological pathways and immune cell functions involved in the allergic immune response, and thus deserve further investigation.

### 2.4. Antimicrobial Activity of Polyphenols

Antibiotic therapy has been a fundamental treatment for skin diseases for many years; however, the adverse reactions caused by medications end up making the treatment unpleasant, in addition to cases of decreased sensitivity to antibiotics. Natural products are becoming increasingly common in dermatology due to the increased resistance of bacteria to synthetic antibiotics and the active principle of medicinal plants becoming new options as antiseptics and antimicrobials. It is believed that flavonoids, such as caffeic acid (CA), benzoic acid, and cinnamic acid, appear to act on the membrane or cell wall of the microorganism, causing functional and structural destruction [65]. Natural polyphenols can play dynamic roles as antimicrobials against bacteria, fungi, and viruses. Pomegranate, a kind of fruit from the Persian region, is rich in polyphenols of varying content during different stages of maturation. Due to its characteristics, pomegranate has medicinal purposes and is used to treat strep throat, hoarseness, and fever, and also has antiviral and antiseptic uses. Pomegranate polyphenols revealed antimicrobial activity when assayed against *Pseudomonas aeruginosa*, *Escherichia coli*, *Candida albicans*, methicillin-resistant *Staphylococcus aureus* (MRSA), *Cryptococcus neoformans*, *Mycobacterium intracellulare*, and *Aspergillus fumigatus* [66]. *Micrococcus luteus* is a kind of non-pathogenic skin commensal bacterium, although it can act as an opportunistic pathogen and cause serious infections, especially for patients with catheters and comorbidities. Pomegranate polyphenols showed antimicrobial activity against *M. luteus* via inhibition of biofilm formation. Grape seed polyphenols have shown effective antimicrobial properties and were efficiently used against Gram-positive bacteria (*Bacillus cereus*, *Staphylococcus aureus*, *Bacillus coagulans*, and *Bacillus subtilis*), but they were more effective against Gram-negative bacteria, such as *P. aeruginosa* or *E. coli* [67]. *Schinus terebinthifolius Raddi* was found to enhance microbial inhibition against the tested strains, especially against Gram-negative bacteria [68]. Its potential use as an alternative to overcome bacterial resistance can be expected. Phlorotannins, polyphenols extracted from brown seaweeds, are recognized for their antimicrobial biological capacity. Phlorotannins were more effective against Gram-positive bacteria, with *Staphylococcus epidermidis* being the most susceptible species [69]. Resveratrol has been shown to inhibit 80% of the growth of dermatophytes of *Trichophyton mentagrophytes*, in particular, and was demonstrated to be an apoptosis inducer in the human pathogenic fungus *C. albicans* by activating metacaspase and promoting cytochrome c release [70]. Pro-anthocyanidins are common natural polyphenols, which were shown to reduce the adherence properties of *C. albicans* by attenuating the inflammatory response and interfering with NF-κB and p65 activation and the phosphorylation of specific signal intracellular kinases [71]. Although the underlying molecular mechanisms of the antimicrobial properties of polyphenols remain poorly understood, existing research results may turn many polyphenols into potent and novel pharmacological alternatives for the treatment of a wide range of microbial infections.

### 2.5. Polyphenols as Anticancer Agents for Skin

Chemotherapy, immunotherapy, radiotherapy, and targeted therapy are included in the current management of metastatic and/or non-metastatic skin cancer. The above methods are highly toxic, expensive, and, in some cases, ineffective due to the development of resistance, especially in metastatic cancer [72]. Thus, it is important to propose new effective therapeutic strategies or drugs which are more affordable and safer. Accumulating evidence from the last decade indicates that promising anticancer natural compounds, such as EGCG, resveratrol, and curcumin, among others, may be extracted from plants [73,74,75,76]. Polyphenols may exert these anticancer effects via a variety of mechanisms, including removal of carcinogenic agents, modulation of cancer cell signaling and cell cycle progression, promotion of apoptosis, and modulation of enzymatic activities. Tea polyphenols are abundant in green tea leaves, accounting for ca. 30% of dry leaf weight, and are also collectively referred to as catechins. The biological bioactivities of tea polyphenols, with EGCG as the primary contributor, have been well documented and include anticancer effects and reduced risk of degenerative diseases. Tea polyphenols can reduce UV-induced mouse skin carcinogenesis in terms of tumor incidence and multiplicity [77]. Tea polyphenols provided protection against 7,12-dimethyl benz(a)anthracene-induced mouse skin tumorigenesis. A population-based case-control study indicated that strong (hot) black tea had independent potentially protective effects against skin squamous cell carcinoma [78]. Pre-clinical trials have examined the anticancer properties of resveratrol in skin [79]. The underlying anticancer mechanisms of resveratrol have been shown to be due to the induction of apoptosis, antioxidant systems, amelioration of inflammation, and cell cycle suppression in mouse skin carcinogenesis models [80,81,82]. Curcumin extracted from *Curcuma longa L.* was also indicated to have anticancer biological activities [83]. 

**Figure 2:**
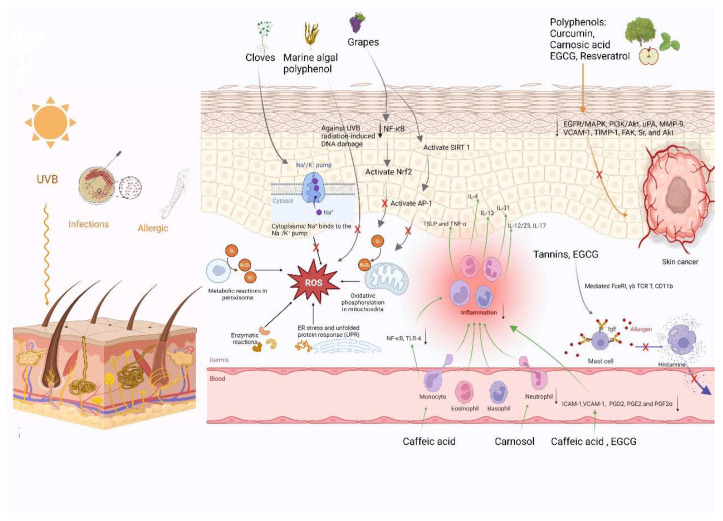
Overview of different mechanisms of several natural polyphenols on skin health.

Several signaling pathways are involved in the mechanism of polyphenols against skin cancer metastasis, including NF-κB, epidermal growth factor receptor/mitogen activated protein kinase (EGFR/MAPK), and phosphatidylinositide 3- kinases/protein kinase B (PI3K/Akt) [83,84]. Carnosic acid has been shown to play an important protective role against melanoma. This secondary metabolite inhibited the adhesion and proliferation of B16F10 melanoma cells in a dose-dependent manner via inhibition of the expression of cell migration markers (uPA, MMP-9, VCAM-1, and TIMP-1) and phosphorylation of signaling molecules (FAK, Sr, and Akt) [85]. A series of studies have demonstrated that various polyphenol-rich fruits and vegetables are particularly effective in protecting against colon cancer development. In general, the anticancer effects of polyphenols are a comprehensive reflection of their anti-inflammatory and antioxidant properties, as well as other effects (Figure 2).

## 3. Roles of Polyphenols in Hair Health

### 3.1. Effects of Polyphenols on Hair Growth 

Alopecia is characterized by the loss of some or all hair. Millions of individuals have suffered due to hair loss resulting from a variety of reasons, including the primary genetic cause, social, psychological, and mental stress, local infection, and endocrine disorders. Hair disorders may considerably impact the social and psychological well-being of an individual. Androgenetic alopecia (AGA) and alopecia areata (AA) are the most common types of hair disorder. AGA affects approximately 50% of men and women. AA occurs in 2% of the population. In general, hair loss may affect up to 70% of men and 50% of women at some point in their lifetime [86]. AGA is associated with high 5-α-reductase activity, elevated 5-α-dihydrotestosterone (DHT), and dysregulated transforming growth factor-β (TGF-β) signaling [87]. Identification of lymphocytic infiltrates in AA lesions gave rise to the hypothesis that there is an autoimmune attack on hair follicles (HFs), which is likely a consequence of loss of immune privilege mediated by immune T cells [88]. 

**Figure 3:**
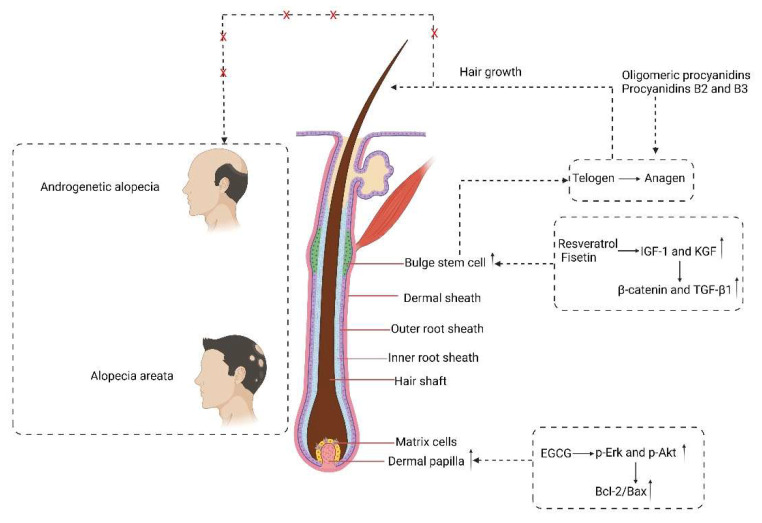
Mechanisms of natural polyphenols in the treatment of human AGA and AA.

It was reported that EGCG might be useful in the prevention or treatment of AGA by selectively inhibiting 5-α-reductase activity [89]. EGCG promoted hair growth in hair follicles in ex vivo culture and the proliferation of cultured dermal papilla cells (DPCs). The growth stimulation of DPCs by EGCG in vitro may be mediated through the upregulation of phosphorylated Erk and Akt and by an increase in the ratio of Bcl-2/Bax [90]. Resveratrol and fisetin regulated the genetic expression of cytokines, such as insulin-like growth factor-1 (IGF-1) and keratinocyte growth factor-2 (KGF), which activate the β-catenin pathway, and TGF-β1, which plays an important role in maintaining the niche of hair follicle stem cells, and were thus thought to play roles in promoting hair growth. Resveratrol and fisetin induced a shift from telogen to anagen in the hair follicle by inducing proliferation of hair follicle bulge stem cells, thus promoting hair growth [91]. Procyanidin has been found to decrease the expression of protein kinase C (PKC) in hair epithelial cells and stimulate anagen induction [92]. Additionally, it is also posited that procyanidin and flavonoids may counteract TGF-β-induced cell death by inhibiting 5-α-reductase, antioxidant-related mechanisms, and upregulating the expression of anti-apoptotic factors, such as Bcl-xL [92]. Oligomeric procyanidins have also shown remarkable hair growth stimulant effects in vitro and in vivo, being able to promote hair epithelial cell growth and anagen induction of the hair cycle [93]. In particular, procyanidins B2 and B3 show evidence of protective action against apoptosis in hair epithelial cell cultures, thereby restricting catagen induction in the hair cycle [94]. All of the above phenolics are expected to play important roles in the treatment of human AGA and AA (Figure 3).

### 3.2. Effects of Polyphenols on Hair Pigmentation

Hair pigmentation is determined by the degree and distribution of melanin in the cortex. Photodamage to hair (photobleaching) may be caused by UVA, UVB, and visible radiation, and the effects of different wavelength ranges vary. UVB and UVA radiation interact negatively with hair proteins, while visible light promotes melanin granule degradation. *Punica granatum L.* hydroalcoholic extract reduced photodamage of hair exposed to UVA radiation [95]. In addition, natural polyphenols, including tannins, present high antioxidant activity and could be used to reduce fading of natural hair color.

Hair dyeing is a common method used to recolor hair. Traditional commercial permanent hair dyeing products usually contain p-phenylenediamine (PPD) or PPD-derived compounds and hydrogen peroxide as key ingredients. These components are reportedly toxic, allergenic, mutagenic, and potentially carcinogenic to people. Recently, a method using metal—phenolic networks (MPNs), such as tannic acid (TA)-based MPNs and gallic acid (GA)-composed MPNs, reportedly dyed natural gray hair without potentially toxic chemicals and protected the dyed hair against repeated shampoo washing [96].

## 4. Summary and Remaining Problems

Polyphenols are ubiquitously found in plants and therefore consumed in relatively high quantities in the human diet. Polyphenolic extracts are attractive ingredients for pharmaceuticals and cosmetics due to their beneficial and multifunctional biological properties and abundant availability in various dietary sources. However, similar to conventional drugs, natural polyphenols can be toxic if they accumulate beyond acceptable levels in the human body. Moreover, some studies reported polyphenols consumed in whole foods; thus, we do not know whether the results are due to interactions between polyphenols and other ingredients, and further research is needed to focus on the isolated forms of natural polyphenols. Indeed, studies investigating the beneficial effects of polyphenols, and the magnitude of the effects, must consider interfering matrix effects, enzymatic interactions, reactions with other foods, and genetic or gender characteristics [97]. In addition, most of the clinical studies exploring isolated forms of polyphenols were short-term studies; long-term health and adverse effects should be elucidated in future studies [98,99,100]. The effects of polyphenols on skin and hair are primarily determined by their physicochemical properties. Therefore, it is important to assess the effectiveness of polyphenolic compounds against skin and hair diseases applied systemically and/or topically. Despite the fact that polyphenols are multi-potent compounds that can be used in the treatment of a wide spectrum of diseases, including skin and hair diseases, some properties may limit their efficient use in therapy, such as low water solubility and poor stability. Improving the percutaneous absorption of plant polyphenols is a direction of future research.

## Figures and Tables

**Table 1:** Several marketed formulations based on polyphenols as anti-aging cosmeceuticals.

Plant	Compounds	Bioactivity
Blackberry	Anthocyanins	↓IL-6, ↓TNF-a, ↓ERK1/2, ↓P38, ↓JNK1/2, ↓MKK4, ↓PGE2, ↓iNOS, ↓NF-кB, ↓Iк-ßa
Cacao bean	Flavonoids	↓Wrinkle formation, ↑Collagen level, ↓MMP-1, ↓AP-1 expression
Strawberry	Phenolic	↓ROS, ↓NF-кß, ↓Iк-ßa phosphorylation, ↓TNF-a, ↓IL-6, ↓IL-1b, ↑Nrf2, ↑CAT, ↑HO-1
Black rice	Flavonoids	↓ROS, ↓MMP-1, ↓MMP-3, ↓Procollagen type 1, ↓p-cfos, ↓p-cjun, ↓p-p38, ↓p-JNK
Grape	Flavonoids Phenolics Anthocyanins Resveratrol	↑Nrf2, ↑HO-1, ↓MMP-1, ↓MMP-9
Tea	EGCG	↑Erk, ↑Akt, ↑Bcl-2/Bax
Clove	Eugenol Gallic acid	↓Skin wrinkle, ↓Skin Thickness, ↓ROS, ↓MMP-1, ↓MMP-3, ↓IL-6, ↓p-c-fos, ↓p-c-jun, ↑NF-кB, ↑Ik-ßa, ↑Nrf2, ↑HO-1, ↑NQO-1, ↑Skin hydration, ↑p-Smad2/3, ↑TGF-ß1

## Data Availability

Not applicable.

## Sample Availability

Samples of the compounds are not available from the authors.
